# The Incidence of Nasopharyngeal Carcinoma in Pahang State of Malaysia from 2012 to 2017

**DOI:** 10.21315/mjms2021.28.1.9

**Published:** 2021-02-24

**Authors:** Azmir Ahmad, Wardah Mohd Yassin, Nor Azlina A Rahman, Wan Ishlah Leman, Luqman Rosla, Mark Paul, Sharifah Nor Ezura Syed Yussof, Kamariah Mohamed@Awang, Kahairi Abdullah, Mohd Arifin Kaderi

**Affiliations:** 1 Department of Basic Medical Science for Nursing, Kulliyyah of Nursing, International Islamic University Malaysia, Pahang, Malaysia; 2 Department of Biomedical Science, Kulliyyah of Allied Health Sciences, International Islamic University Malaysia, Pahang, Malaysia; 3 Department of Otorhinolaryngology-Head and Neck Surgery, Kulliyyah of Medicine, International Islamic University Malaysia, Pahang, Malaysia; 4 Department of Otorhinolaryngology, Hospital Sultan Haji Ahmad Shah, Pahang, Malaysia; 5 Department of Otorhinolaryngology, Hospital Tengku Ampuan Afzan, Pahang, Malaysia; 6 Ear, Nose and Throat Consultant, KPJ Batu Pahat Specialist Hospital, Johor, Malaysia

**Keywords:** nasopharyngeal carcinoma, incidence of nasopharyngeal carcinoma, Malaysia, incidence rate, crude, age-standardised rate

## Abstract

**Background:**

Nasopharyngeal carcinoma (NPC) is the fifth most common cancer among Malaysians. While several studies have reported the trend of NPC in other states in Malaysia, no studies have reported the trend of NPC in Pahang state. This study was designed to report the number and distribution of newly diagnosed NPC cases in Pahang.

**Methods:**

NPC cases that were diagnosed between 2012 and 2017 in two referral hospitals in Pahang were traced. The crude incidence rate (CR) and age-standardised rate (ASR) were calculated to investigate the NPC incidence.

**Results:**

There were 143 new cases of NPC reported from the two hospitals. The mean age at diagnosis was 52.0 ± 13.7 years old. The majority of cases involved males (74.1%) with a male to female ratio of 2.9:1. Chinese males were found to have the highest incidence with a mean ASR of 4.7 per 100,000 population. Overall, the mean ASR for Pahang was 2.4 per 100,000 population for males and 0.9 per 100,000 population for females.

**Conclusion:**

The total number of NPC cases reveals an increasing trend from 2012 to 2014 and then a slightly decreasing trend from 2015 to 2017. The incidence of NPC in Pahang was intermediate in males and low in females.

## Introduction

Nasopharyngeal carcinoma (NPC) is a type of malignant tumour of the nasopharynx that arises from the mucosal epithelium. Globally, NPC is considered a rare malignancy with an incidence rate below 1 per 100,000 persons per year for both genders. In 2012, NPC ranked as the 24th most common new cancer in the world with 86,691 cases and 50,831 deaths (1). Notably, NPC is a disease with remarkable geographic and racial distribution worldwide in which it is prevalent in certain regions, such as Southern China, Southeast Asia, North Africa and the Arctic.

Based on data from the Cancer Incidence in Five Continents, the highest rate according to region was demonstrated in a population in Southeast Asia that had an overall incidence of 6.4 per 100,000 person-years among males and 2.4 per 100,000 person-years among females. A high incidence of NPC was also found in populations in Micronesia/Polynesia, Eastern Asia and Northern Africa, where incidence rates were above 2.0 per 100,000 person-years in males and 1.0 per 100,000 person-years in females. Specifically, the high incidence areas are centralised in the southern part of China, particularly Guangdong Province, with approximately 20 to 40 cases per 100,000 person-years depending on the region (2). Cities in Guangdong Province, namely Zhongshan, Guangzhou and Sihui, had a high incidence in males compared to elsewhere in the world, with an age-standardised rate (ASR) of 26.8 per 100,000 person-years (3), 22.2 per 100,000 person-years (4) and 30.94 per 100,000 person-years (5), respectively. In addition, a high incidence of this tumour was observed in nearby cities in Southern China, including Hong Kong and Macao, with ASRs of 14.4 and 15.7 per 100,000 person-years among males, respectively (2).

Intermediate rates have been reported in Southeast Asian countries, such as Singapore, Malaysia, the Philippines, Thailand, Indonesia and Vietnam, where the ASR ranges from 3.4 to 12.9 per 100,000 person-years for males. Similarly, in Northeast India, intermediate ASRs were recorded for males in Mizoram and Sikkim states (5.0 and 3.6 per 100,000 person-years, respectively). Other than Asia, intermediate incidence of NPC has been reported in North Africa (Algeria, Tunisia and Libya), where it ranges from 1.0 to 6.0 per 100,000 person-years (2).

In Malaysia, NPC is a significant health burden, as it is among the five most commonly diagnosed cancers in the country’s population and fifth most common cancer among Malaysian men (6). Malaysian National Cancer Registry Report 2012–2016 discovered that there were on average 900–1,000 cases of NPC reported each year in 2007–2011. The incidence was more than twofold higher among males compared to females. The ASR for males was 6.4 per 100,000 population, whereas it was 2.2 per 100,000 population for females. According to ethnicity, Chinese males were found to have a higher incidence with an ASR of 11.0 per 100,000 population compared to Malay males (3.3 per 100,000 population) and Indian males (1.1 per 100,000 population). Of specific interest, the incidence is particularly high among some native groups in Sarawak. In 1996–1998, an ethnic-based study on NPC revealed that a native group in Sarawak (Bidayuh) showed the highest rate of NPC (31.5 per 100,000 population in males) compared to other ethnicities in Malaysia or any other ethnicity worldwide (7).

NPC is a rare cancer worldwide, but it is common in certain geographic regions, including in Malaysia. It is necessary to determine the incidence and identify the affected groups. The existing data are limited, as research regarding this disease has been conducted in certain states only. There are limited updated NPC statistics in Pahang state of Malaysia. Basic information on NPC in Pahang is crucially needed before further research on the disease in the region can be carried out. Thus, this epidemiological study is designed to feature the number of cases and distribution of NPC in Pahang.

## Methods

### Data Collection

This study involved two main referral hospitals in Pahang, namely Hospital Tengku Ampuan Afzan (HTAA) in Kuantan and Hospital Sultan Haji Ahmad Shah (HOSHAS) in Temerloh. It included newly diagnosed cases of NPC reported from 1 January 2012 until 31 December 2017 from both hospitals. Newly diagnosed cases of NPC were defined as patients who were diagnosed with NPC for the first time and not recurrent cases. This was confirmed by reviewing the patients’ medical records from both hospitals. All records were traced from the Cancer Notification Registry from the Department of Otorhinolaryngology — Head and Neck Surgery. Each patient’s information such as name, gender, address, identification number, date of diagnosis and type and stage of NPC were collected from the records. Only Malaysian citizens who had been diagnosed with NPC in Pahang were included in the data analysis. The histopathological classification was based on the World Health Organization (WHO) classification of NPC (8). The tumours were staged according to the American Joint Committee on Cancer staging for NPC (2002) (9).

### Calculation of Crude Incidence Rate and Age-Standardised Rate

The incidence of NPC was computed based on the number of individuals in the total population who were newly diagnosed with NPC in Pahang for 2012–2017. The formula for the calculation of the crude incidence rate (CR) and ASR were taken from the Malaysian National Cancer Registry Report 2012–2016 (6). CR is the number of newly diagnosed cases of NPC observed in the Pahang population during the study period (2012–2017) divided by the total at-risk Pahang population number in the same period, as follows:

$$CR-\frac{N}{P}\times 100,000$$

where *N* is the total number of newly diagnosed cases of NPC and *P* is person-years at risk (6). These statistics can be obtained from the database of the Department of Statistics Malaysia for the years 2012 until 2017 (10). The CR was expressed per 100,000 population. To note, in the database of the Department of Statistics Malaysia, the Malay and indigenous populations are grouped as the Bumiputera group. Therefore, in this study, the CR and ASR for the Malay and indigenous populations were reported together as the Bumiputera group.

Cancer is a disease that relies on the age structure of the population. As CR does not take into account the varying age structures in the underlying populations, ASR was used for meaningful comparisons of two populations. Before determining the ASR, the incidence rates in specified five-year age groups, starting from 10 to 15 years old until 80 to 85 years old, were calculated. This is known as the age-specific rate (AR). It was calculated by dividing the number of newly diagnosed cases of NPC in the five-year age groups by the total number of Pahang population in the particular age group and multiplying by 100,000 population, as follows:

$$AR-\frac{N_{i}}{P_{i}}\times 100,000$$

where *N_i_* is the number of new cancers occurring in the *i*th age group and *P_i_* is at-risk person-years in the *i*th age group (6). This can be obtained from the database of the Department of Statistics Malaysia for the years 2012 until 2017 (10).

Next, the ASR was calculated by summing the AR weighting to the world standard population (6), as follows:

$$ASR=\frac{\sum \left({AR}_{i}\times {\text{P}}_{i.std}\right)}{Total\;world\;standard\;population}$$

where *AR_i_* is the age-specific rate in the *i*th age class and *P_i.std_* is the number in the *i*th age class of the world standard population (6). The calculated ASR was then called the world standardised incidence rate and also expressed per 100,000 population. The world standard population that was modified by Doll et al. (11) was applied as a reference in calculating the ASR, as shown in Table 1. The world standard population is used for age adjustment purposes in calculating the ASR to make age-specific comparisons of one population with the world population more meaningful statistically (12). The incidence of NPC by gender and ethnicity was identified as the result of the calculation. All the data were computed using Microsoft Office Excel 2013.

## Results

### Distribution and Clinical Presentation of NPC Patients in Pahang from 2012 until 2017

A total of 143 newly diagnosed cases of NPC were reported in Pahang throughout the years 2012–2017. The data came from HTAA in Kuantan (60.8%) and HOSHAS in Temerloh (39.2%). The age at diagnosis of the patients ranged from 14 to 82 years old with a mean age of 52.0 ± 13.7 years old. The majority were male (74.1%) with a male to female ratio of 2.9:1. Regarding ethnicity, Malay predominated with 59.4%, followed by Chinese (35%) and indigenous (5.6%). Table 2 presents the distribution of NPC cases in Pahang by gender and ethnicity.

The distribution of NPC patients in Pahang according to clinical presentation is shown in Table 3. Sixty-eight percent (*n* = 97) of the new cases were undifferentiated carcinomas (WHO type III), while most of the newly diagnosed patients (58.8%) presented at late stages (stages III and IV).

**Figure 1:**
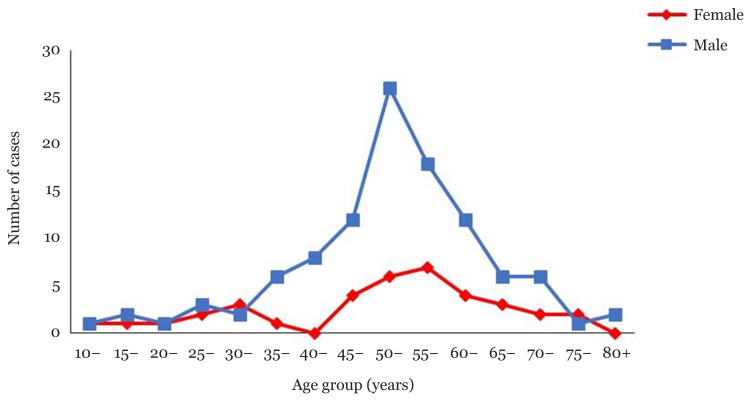
Number of newly diagnosed NPC cases by age group and gender in Pahang, 2012–2017

Figure 1 illustrates the number of NPC cases in Pahang according to age group and gender. From the chart, more males were found to have NPC compared to females. The youngest patients were diagnosed as early as 14 years old in males and females. Generally, the number of cases for both genders demonstrated a rising number of cases with each age increment up to 55 years old, followed by a decreasing trend onwards. The peak number of NPC cases for male patients was in the 50–54 years old age group with 26 cases. The highest number for female patients was in the 55–59 years old age group with seven cases. After reaching the peak number, there was a decline of cases for both genders until the age of 82 years old in males and 75 years old in females.

### Incidence of NPC in Pahang from 2012 until 2017

The actual number of cases and the CR and ASR per 100,000 population of newly diagnosed NPC cases by ethnic group and gender are presented in Table 4. Table 4 shows that males from both ethnicities had higher ASRs compared to females. In 2014, Chinese males were found to have the highest ASR of 7.0 per 100,000 population. Meanwhile, Bumiputera males had the highest ASR of 3.5 per 100,000 population in 2015. In females, Bumiputera had the highest ASR of 1.5 per 100,000 population in 2017, whereas Chinese had 1.2 per 100,000 population in 2014.

On the other hand, the lowest ASRs for males were 1.4 per 100,000 population among the Bumiputera group and 1.3 per 100,000 population in the Chinese population. In females, the Bumiputera group had the lowest ASR in 2015 with 0.7 per 100,000 population. For Chinese females, there were no cases reported in 2013 and 2017. The lowest ASR for this group was reported in 2016 with 0.5 per 100,000 population.

The mean CR and ASR for the Bumiputera group and Chinese ethnicities are summarised in Table 5. Chinese males were found to have the highest incidence with a mean ASR of 4.7 per 100,000 population. The risk in Chinese males was nearly twofold higher than it was in the Bumiputera males. In contrast, Chinese females were recorded as the lowest incidence with a mean ASR of 0.6 per 100,000 population. For the Bumiputera group, the mean ASRs were 1.9 per 100,000 population and 1.0 per 100,000 population in males and females, respectively. Overall, the ASRs for Pahang were 2.4 per 100,000 population in males and 0.9 per 100,000 population in females.

## Discussion

There were 143 newly diagnosed cases of NPC reported in Pahang over the study period with an average of 24 cases identified every year. This figure only reflects the cases reported at the government hospitals. In comparison with the Malaysia National Cancer Registry Report 2012–2016, 221 cases of NPC were reported in Pahang, which included the records from both government and private hospitals (6). The overall male to female ratio was 2.9:1. Globally, the male to female ratio for most populations ranges from 2–3:1 (4). The higher incidence among males may reflect different lifestyles, such as exposure to smoking, as well as genetic considerations.

This study analysed NPC patients from three ethnic groups: Malay, Chinese and Indigenous. Malay and Indigenous were categorised as the Bumiputera group. Among these ethnic groups, Chinese accounted for the highest incidence (mean ASR 4.7 per 100,000 population in males) of NPC cases in Pahang. The latest cancer registry reported that the NPC incidence in Malaysia was high among the Chinese, intermediate among Malays and low among Indians (6). NPC is particularly common in certain ethnic groups, especially Chinese, as evidenced by the high rates reported in Southern China and Southeast Asian countries. A previous study discovered a strong genetic influence of human leukocyte antigens-A in Malaysian Chinese that contributed to NPC susceptibility (13).

In addition, the distribution of the pathological type of NPC varies in different regions. Data from the current study demonstrate that the majority of NPC patients with known histological status were diagnosed with WHO type III (67.5%), and 58.8% of the cases presented with late stage at diagnosis. Notably, WHO type III NPC accounted for more than 97% of cases in endemic areas like Southeast Asia, while WHO type I was found more often in Western countries (14). Thus, the pathological type of NPC cases in Pahang seems to follow the same patterns as elsewhere in Malaysia (15), as well as in Southeast Asian regions (16–18).

Several studies in Malaysia have shown similarly high percentages of late stage at presentation, including in Sarawak (19), Pulau Pinang (20) and Malaysia (6) in general. Among the reasons for this late stage presentation include delays in seeking medical advice, the confusing nature of the presenting symptoms, the difficult nature of clinically examining the nasopharynx and the presence of submucosal lesions with normal appearance during examination (21).

Pahang is the largest state in Peninsular Malaysia. It is located in the east coast region together with Terengganu and Kelantan. In general, this region is relatively less developed than the western part of the Peninsula, with the main economic activities being in agriculture, fisheries and small-scale industries. Data from the Malaysia National Cancer Registry Report 2012–2016 indicated that NPC was less common in this region compared to other parts of the country. Within that period, Pahang was ranked as the tenth state in Malaysia in terms of NPC incidence. However, no detailed incidence of NPC cases by state on gender and ethnicity was described in the report (6). This study found that the incidence of NPC by gender in Pahang was intermediate in males (mean ASR 2.4 per 100,000 population) and low in females (mean ASR 0.9 per 100,000 population). The possible explanation is that this state has a higher proportion of Malay residents than Chinese residents.

There were some limitations identified in this study. It was designed specifically to investigate certain aspects of NPC epidemiology in the single state of Pahang. Findings from this study may not be representative of all states or even the entire east coast region of Peninsular Malaysia. Secondly, the data of newly diagnosed NPC cases were collected from the main government hospitals in Pahang. The cases from private hospitals were not included, as the study would require more ethical consideration with the involved centres. Thirdly, despite having collected the data from the main referral hospitals in Pahang, the data for patients living in the Cameron Highlands district were uncertain, as there were no cases reported from that area. This is probably because patients from that district were referred to the nearby hospital located in Perak. Hence, the findings of this study only represent the newly diagnosed NPC cases reported in the main referral hospitals in Pahang and do not include all residents of the state.

## Conclusion

The current study serves as the first study to investigate the epidemiological information on NPC in Pahang. NPC is a disease with unique epidemiological features. The distribution of the disease demonstrates a distinctive regional, racial and gender prevalence. The study showed that the incidence in Pahang was intermediate in males (ASR of 2.4 per 100,000 population), whereas it was low in females (ASR of 0.9 per 100,000 population). Ethnic Chinese were found to have higher rates compared to the Bumiputera group. Overall, based on the total annual number of reported NPC cases in Pahang, there was an increasing trend from 2012 until 2014 and a slowly decreasing trend from 2015 to 2016.

Based on these findings, a longer duration of survey is needed in order to understand the exact trend of NPC occurrence in the state. This can be achieved by establishing an effective cancer registry in the country that includes all cancer cases in every state in Malaysia.

## Figures and Tables

**Table 1:** World standard population modified by Doll et al. (11)

Age class index(i)	Age class	Population (w_i_)
1	0–4	12,000
2	5–9	10,000
3	10–14	9,000
4	15–19	9,000
5	20–24	8,000
6	25–29	8,000
7	30–34	6,000
8	35–39	6,000
9	40–44	6,000
10	45–49	6,000
11	50–54	5,000
12	55–59	4,000
13	60–64	4,000
14	65–69	3,000
15	70–74	2,000
16	75–79	1,000
17	80–84	500
18	85+	500

Total		100,000

**Table 2:** Distribution of NPC cases in Pahang, 2012–2017

Variables		Number	%
Gender	Male	106	74.1
	Female	37	25.9
Ethnicity	Malay	85	59.4
	Chinese	50	35.0
	Indigenous	8	5.6

**Table 3:** Clinical presentation of NPC at diagnosis in Pahang, 2012–2017

Variables		Number	%
Histological types	WHO type I	3	2.1
	WHO type II	25	17.5
	WHO type III	97	67.8
	Unknown	18	12.6
Staging	Stage I	21	14.7
	Stage II	17	11.9
	Stage III	26	18.2
	Stage IV	58	40.6
	Unknown	21	14.7

**Table 4:** Numbers of cases, CR and ASR per 100,000 population of newly diagnosed NPC by ethnic group and gender in Pahang, 2012–2017

Year	Ethnic	Male			Female		
	
		Number	CR	ASR	Number	CR	ASR
2012	Bumiputera	8	1.3	1.6	6	1.1	1.0
	Chinese	2	1.6	1.3	1	0.9	0.7
2013	Bumiputera	8	1.3	1.4	4	0.7	0.8
	Chinese	9	7.4	6.3	0	0	0
2014	Bumiputera	9	1.5	1.8	6	1.0	1.0
	Chinese	11	9.0	7.0	2	1.8	1.2
2015	Bumiputera	18	2.9	3.5	3	0.5	0.7
	Chinese	5	4.1	3.3	1	0.9	0.9
2016	Bumiputera	11	1.8	1.9	4	0.7	0.8
	Chinese	9	7.4	4.8	1	0.9	0.5
2017	Bumiputera	7	1.1	1.4	9	1.5	1.5
	Chinese	9	7.4	5.4	0	0	0

**Table 5:** Total numbers of cases, mean of CR and ASR per 100,000 population of newly diagnosed NPC by group and gender in Pahang, 2012–2017

Group	Male			Female		
	
	Number	CR	ASR	Number	CR	ASR
Bumiputera	61	1.6	1.9	32	0.9	1.0
Chinese	45	6.2	4.7	5	0.7	0.6

Total	106	2.3	2.4	37	0.7	0.9
